# Human Prostate Cancer Is Characterized by an Increase in Urea Cycle Metabolites

**DOI:** 10.3390/cancers12071814

**Published:** 2020-07-06

**Authors:** Andras Franko, Yaping Shao, Martin Heni, Jörg Hennenlotter, Miriam Hoene, Chunxiu Hu, Xinyu Liu, Xinjie Zhao, Qingqing Wang, Andreas L. Birkenfeld, Tilman Todenhöfer, Arnulf Stenzl, Andreas Peter, Hans-Ulrich Häring, Rainer Lehmann, Guowang Xu, Stefan Z. Lutz

**Affiliations:** 1 Department of Internal Medicine, Division of Endocrinology, Diabetology and Nephrology, University Hospital Tübingen, 72076 Tübingen, Germany; andras.franko@med.uni-tuebingen.de (A.F.); martin.heni@med.uni-tuebingen.de (M.H.); andreas.birkenfeld@med.uni-tuebingen.de (A.L.B.); hans-ulrich.haering@med.uni-tuebingen.de (H.-U.H); s.lutz@bad-sebastiansweiler.de (S.Z.L.); 2 Institute for Diabetes Research and Metabolic Diseases of the Helmholtz Centre Munich at the University of Tübingen, 72076 Tübingen, Germany; 3 German Center for Diabetes Research (DZD), 72076 Tübingen, Germany; 4 CAS Key Laboratory of Separation Science for Analytical Chemistry, Dalian Institute of Chemical Physics, Chinese Academy of Sciences, Dalian 116023, China; alanna_s@foxmail.com (Y.S.); hucx@dicp.ac.cn (C.H.); liuxy2012@dicp.ac.cn (X.L.); xj_zhao1@126.com (X.Z.); wangqq15@dicp.ac.cn (Q.W); 5 Department of Urology, University Hospital Tübingen, 72076 Tübingen, Germany; joerg.hennenlotter@med.uni-tuebingen.de (J.H.); todenhoefer@studienurologie.de (T.T.); arnulf.stenzl@med.uni-tuebingen.de (A.S.); 6 Institute for Clinical Chemistry and Pathobiochemistry, Department for Diagnostic Laboratory Medicine, University Hospital Tübingen, 72076 Tübingen, Germany; miriam.hoene@med.uni-tuebingen.de (M.H.); andreas.peter@med.uni-tuebingen.de (A.P.); 7 Clinic for Geriatric and Orthopedic Rehabilitation Bad Sebastiansweiler, 72116 Mössingen, Germany

**Keywords:** prostate cancer, metabolomics, urea cycle, fumarate, oncometabolite, NFκB

## Abstract

Despite it being the most common incident of cancer among men, the pathophysiological mechanisms contributing to prostate cancer (PCa) are still poorly understood. Altered mitochondrial metabolism is postulated to play a role in the development of PCa. To determine the key metabolites (which included mitochondrial oncometabolites), benign prostatic and cancer tissues of patients with PCa were analyzed using capillary electrophoresis and liquid chromatography coupled with mass spectrometry. Gene expression was studied using real-time PCR. In PCa tissues, we found reduced levels of early tricarboxylic acid cycle metabolites, whereas the contents of urea cycle metabolites including aspartate, argininosuccinate, arginine, proline, and the oncometabolite fumarate were higher than that in benign controls. Fumarate content correlated positively with the gene expression of oncogenic HIF1α and NFκB pathways, which were significantly higher in the PCa samples than in the benign controls. Furthermore, data from the TCGA database demonstrated that prostate cancer patients with activated NFκB pathway had a lower survival rate. In summary, our data showed that fumarate content was positively associated with carcinogenic genes.

## 1. Introduction

With 1.4 million cases in 2016, prostate cancer (PCa) was the most common incident of cancer among men [1]. Current therapeutical options for the treatment of localized PCa include radical prostatectomy, radiation, and androgen-deprivation therapies [2]. PCa is flexible and often becomes resistant to androgen deprivation therapy, in the course of treatment [3]. PCa is also frequently associated with such metabolic disarrangements as obesity, metabolic syndrome, and diabetes [3]. The association between PCa and diabetes is complex. Albeit a negative association is observed between patients with late-stage diabetes and prostate cancer, PCa patients with diabetes are characterized by more aggressive cancer than non-diabetic patients [3,4,5].

An understanding of the cellular metabolism of cancer cells is essential in the development of new therapeutical approaches [6]. Cellular metabolism is a complex process, in which nutrients are taken up by the cells and metabolized via interconnected pathways into small molecules called metabolites. In the course of oncogenic transformation, normal cells transform to cancer cells, which try to modify cellular growth, override death signals, induce invasion, initiate angiogenesis and escape the immune system [2]. These versatile molecular changes require an altered cellular metabolism—known as metabolic transformation—to support the adaptation of cancer cells to the novel transformed state [2,7]. Unlike other tumor cells, PCa cells are characterized by unique metabolic transformations in terms of glycolysis, fatty acid metabolism, and tricarboxylic acid (TCA) cycle, since the energy-inefficient healthy epithelial prostate cells are transformed to energy-efficient PCa cells [7]. Furthermore, glucose and fatty acid metabolism are reported to differ between early and late stages of PCa, indicating that metabolic pathways play a central role during tumor progression [2].

Deciphering the metabolic alterations of PCa could pave the way for the identification of new biomarkers [8] and metabolomic approaches such as NMR and mass spectrometry are powerful tools in this respect [9]. The study of Hahn and colleagues revealed that metabolites such as citrate, glutamate and taurine have the diagnostic potential to differentiate PCa from benign tissue [10]. Swanson and co-workers found that benign prostate tissue could be differentiated from PCa samples, on the basis of choline-containing compounds, polyamines and citrate levels [11]. In comparison to benign controls, PCa tissue was reported to contain high levels of lactate [12]. In addition to being used as a potential biomarker for PCa, an altered metabolite pattern in PCa patients is implicated in tumor progression. Lipidomic profiling of plasma samples from castration-resistant prostate cancer patients determined that sphingolipids were associated with a poor prognosis [13]. In PCa samples, glycerophosphorylcholine and phosphorylcholine to creatine ratio was correlated with KI67 tumor proliferation marker [14]. These results suggest that the altered metabolite pattern of patients with PCa could contribute to cancer progression.

Mitochondria play a crucial role in the cellular metabolism of PCa [7]. PCa tissue was characterized by an altered pattern of mitochondrial metabolites [15,16]. D-2-hydroxyglutarate, L-2-hydroxyglutarate, succinate, and fumarate were all recently identified as oncometabolites, which are possible intermediates of the mitochondrial TCA cycle [17]. The accumulation of these oncometabolites was shown to drive cancer progression [18]. Fumarate can activate the oncogenic hypoxia-inducible factor (HIF) pathway, via the inhibition of 2-oxygenase superfamily [19]. This superfamily contains TET proteins and histone demethylase enzymes, both of which are major epigenetic regulators [20]. In renal cancer, fumarate induces HIF1α pathway via nuclear factor ’kappa-light-chain-enhancer’ of the activated B-cells (NFκB) signaling [21]. In addition to the activation of the HIF1α pathway, the accumulation of fumarate also results in increased protein succination, which is involved in antioxidant signaling controlled by nuclear factor-like 2 (NRF2) activation [19]. DeNicola and colleagues showed that tumorigenesis in mice was induced by NRF2 signaling [22], which is postulated to promote cell survival [19]. These results indicate that the HIF1α and NFκB pathways play a pivotal role in fumarate-induced carcinogenesis. 

To investigate key metabolites including mitochondrial pathways that potentially contribute to the development of PCa, we applied a comprehensive, combined metabolomics profiling, using capillary electrophoresis (CE) and liquid chromatography (LC), coupled with mass spectrometry (CE–MS and LC–MS). This mass spectrometry approach enabled us to gain comprehensive, relative quantification of cellular metabolites, including mitochondrial metabolites. In view of the fact that the metabolism differed at various tumor stages, we specifically selected patients at a similar tumor stage and with comparable ISUP 2014/WHO 2016 grades and comparable PCa areas. PCa samples from these patients were compared to autologous tumor-adjacent benign prostatic tissue (ABT). Furthermore, the expression of the main oncogenic genes from the extended cohorts of PCa and benign prostatic tissues (BT) controls was studied using real-time PCR. 

## 2. Results

**Figure 1:**
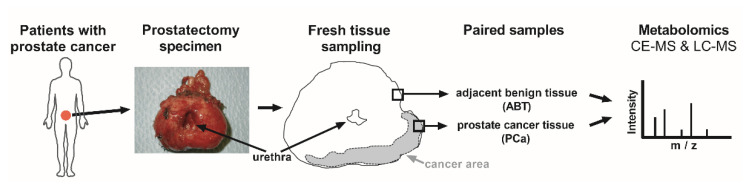
Study workflow. Prostate glands were removed from patients with prostate cancer (PCa), through radical prostatectomy. Anticipated PCa tissue and adjacent benign tissue (ABT) were freshly frozen. Confirmative hematoxylin and eosin staining was later applied on paraffin sections, to verify the histological assignment. Metabolites were isolated from frozen, paired PCa and ABT samples, and were subjected to capillary electrophoresis (CE) and liquid chromatography (LC), coupled with mass spectrometry (MS).

Since the mitochondrial function plays a pivotal role in cancer progression of PCa [7], we concentrated on the comparison of the mitochondrial metabolite patterns of PCa, with adjacent benign paired prostate tissue (ABT) controls (Figure 1).

To investigate patients with a comparable tumor status, thirteen PCa patients with grade 2 or grade 3 (ISUP 2014/WHO 2016 grading [23]) participated in this analysis and were referred to as the “mass spectrometry cohort” (Table 1).

**Figure 2:**
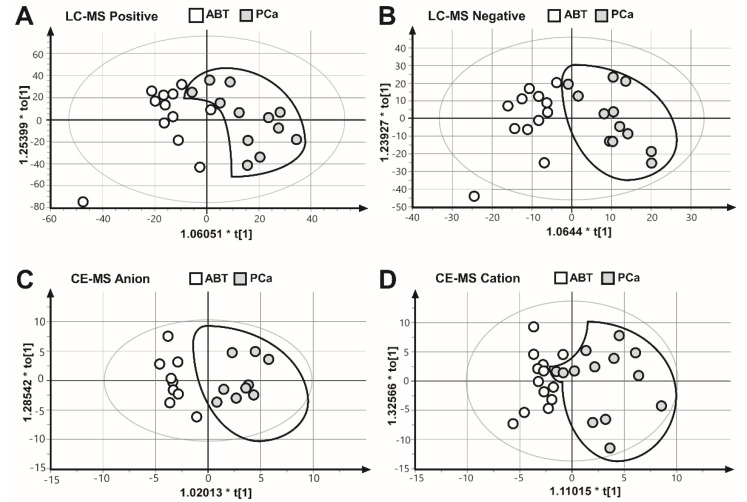
Comparison of the metabolite fingerprints of prostate cancer (PCa) and adjacent benign tissues (ABT) through orthogonal partial least squares discriminate analysis (OPLS–DA). OPLS–DA scores plot of (**A**) LC–MS ESI positive mode R2X = 0.394, R2Y = 0.648, Q2 = 0.189. (**B**) LC–MS ESI negative mode, R2X = 0.250, R2Y = 0.779, Q2 = 0.0649. (**C**) CE–MS anion mode, R2X = 0.247, R2Y = 0.875, Q2 = 0.581. (**D**) CE–MS cation mode, R2X = 0.372, R2Y = 0.663, Q2 = 0.136.

To collect as many mitochondrial metabolites as possible, as well as to gain a comprehensive coverage of nonpolar and polar metabolites, we applied two complementary analysis platforms: LC–MS (positive and negative mode) and CE–MS (anion and cation mode). The results of the four measurements were subjected to OPLS–DA multivariate analysis, to evaluate all measured metabolite values for each biological replicate. These results showed that the PCa samples were separated from the ABT controls (Figure 2), suggesting that benign prostatic tissue and prostate cancer samples are characterized by different metabolic patterns. 

**Figure 3:**
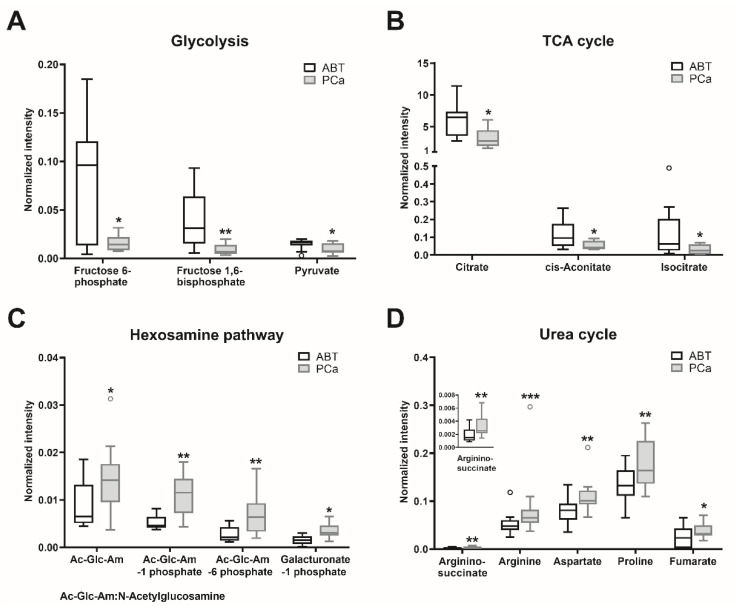
Major metabolites with significant differences between PCa and ABT. Metabolites of prostate cancer (PCa) and adjacent benign tissue (ABT) were measured using CE–MS and LC–MS. Statistical significance was calculated using Wilcoxon signed-rank test and was considered as *p* < 0.05. * *p* < 0.05, ** *p* < 0.01, *** *p* < 0.001. Data are depicted as Tukey boxplots, n: 9–13; raw data is shown in Tables S1–S4. The PCa/ABT ratios, *p*-values and the applied methods are provided in Table S5.

Of all detected metabolites (Tables S1–S4), the major significantly altered metabolites were selected (Table S5). Metabolites involved in amino acid (cysteine, lysine, methionine, phenylalanine, tyrosine, branched-chain amino acids, and protein catabolism) and the lipid (glycerophospholipid) metabolism were enriched in the PCa samples, compared to the ABT controls (Table S5). Since oncometabolites can be synthetized and metabolized via the tricarboxylic acid (TCA) cycle [18], we also analyzed those metabolites involved in the TCA cycle and its adjacent pathways. The contents of glycolytic metabolites fructose 6-phosphate, fructose 1,6-bisphosphate, and pyruvate were significantly lower in the PCa tissues than in the ABT controls (Table S5, Figure 3A). Citrate, cis-aconitate, and isocitrate, which belong to the early steps of the TCA cycle, showed significantly lower contents in PCa than in the ABT samples (Table S5, Figure 3B). Nevertheless, in the PCa samples, the contents of N-acetylglucosamine, N-acetylglucosamine 1-phosphate, N-acetylglucosamine 6-phosphate, and galacturonate 1-phosphate, which belong to the glycolysis connected hexosamine pathway, were significantly increased (Table S5, Figure 3C). Furthermore, contents of metabolites in the urea cycle (aspartate, argininosuccinate, arginine, proline, and fumarate) were significantly higher in PCa than in benign tissue (Table S5, Figure 3D). 

**Figure 4:**
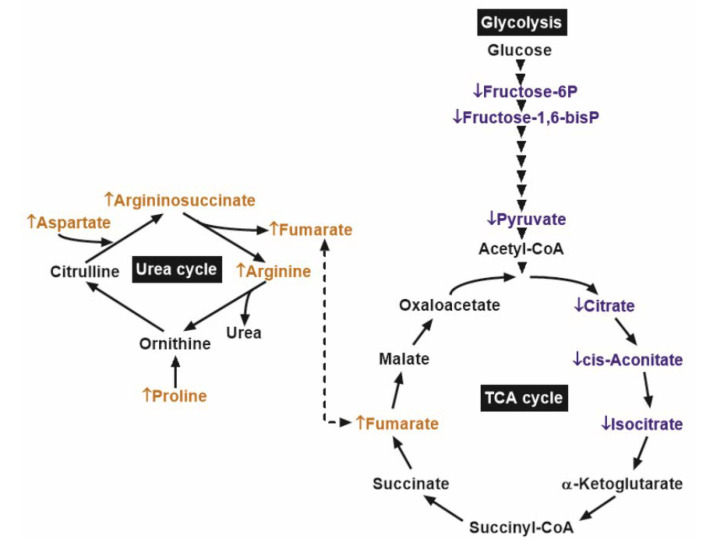
Main biochemical pathways with significant differences between prostate cancer (PCa) and adjacent benign tissues (ABT). ↓ and the blue colors depict metabolites with significantly lower content, while ↑ and the orange colors depict those with significantly higher content, in comparison of PCa to ABT. Arrowheads denote glycolytic enzyme steps.

These results indicated that the reduced glycolytic metabolites in PCa might be associated with lower early TCA cycle intermediates. However, in PCa samples, the concentration of the oncometabolite fumarate was higher than that in benign prostatic tissue, possibly due to replenishment from the urea cycle (Figure 4).

Since fumarate was shown to induce carcinogenesis in various cancer conditions [19], we analyzed the transcript levels of the oncogenic genes, which could be induced by elevated fumarate content. For this real-time PCR analysis, we used age and BMI-matched prostate samples of the extended cohorts, due to the fact that some of the paired prostate samples were completely depleted for the mass spectrometry analysis. These cohorts are known as “extended cohorts”, which involved in one hand new patient samples with benign prostatic tissue (BT) and in the other hand several PCa samples, which were measured by mass spectrometry extended with new PCa samples from other patients. The metabolic parameters of the patients were similar in both groups of the extended cohort (Table 2) and were comparable to the parameters of the patients, whose samples were measured with mass spectrometry (Table 1). 

Abbreviations. BMI: body mass index; BT: benign prostatic tissue; HbA1c: glycated hemoglobin A1c; OGTT: oral glucose tolerance test derived insulin sensitivity index was determined in accordance with Matsuda et al 1999 [24]; and PCa: prostate cancer. Data are given as mean ± standard deviation. To calculate the *p*-values, Mann–Whitney U tests were applied.

**Figure 5:**
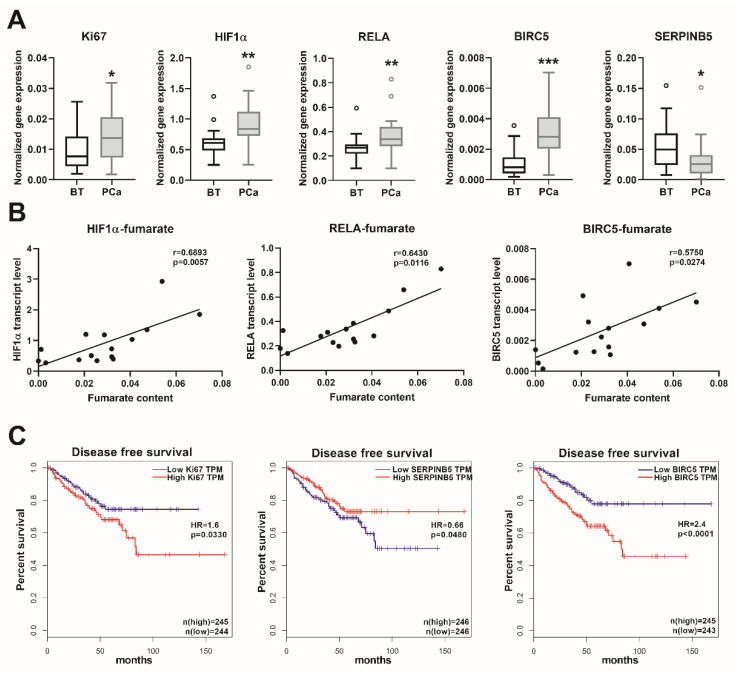
Transcript levels of the oncogenic genes and patients´ survival. (**A**) Transcript levels of the indicated genes were measured using real-time PCR in extended prostate cancer (PCa) and benign prostatic tissue (BT) cohorts. Data are shown as Tukey boxplots, n: 21–23. Statistical significance was calculated using Mann–Whitney U tests and was considered as *p* < 0.05. * *p* < 0.05, ** *p* < 0.01, *** *p* < 0.001. (**B**) The correlation between the fumarate content and the expression of the indicated genes are shown as linear regression (black line). For this analysis, we used those prostate samples from which both fumarate and real-time PCR data were available. Correlation was determined using Spearman’s correlation (see r and *p* values) and statistical significance was considered as *p* < 0.05. (**C**) Disease-free survival rates (Kaplan-Meier curves) of patients with low and high expression for the indicated genes from the human cancer genome atlas (TCGA) (https://www.cancer.gov/tcga). Data were stratified according to the median values (low and high expression groups) and *p*-values were calculated with the log-rank test. HR: hazard ratio, HIF1α: hypoxia-inducible factor 1-subunit alpha, RELA: NFκB subunit RELA proto-oncogene/p65, BIRC5: baculoviral IAP repeat containing 5/survivin, SERPINB5: serpin family B member 5/maspin, and TPM: transcripts per kilobase million.

We observed a significantly higher mRNA expression of the proliferation marker *Ki67* in the PCa samples, in comparison to the BT controls (Figure 5A). The transcript levels of the major regulator of hypoxia-inducible factor 1-subunit alpha (*HIF1α*) and the NFκB subunit *RELA* proto-oncogene/p65 were also significantly higher in the PCa samples (Figure 5A). Furthermore, the transcript levels of the NFκB target genes baculoviral IAP repeat containing 5/survivin (*BIRC5*) and serpin family B member 5/maspin (*SERPINB5*) were significantly up- and downregulated (Figure 5A), respectively, indicating an activated NFκB signaling. None of the other NFκB target genes (*SOCS2, SOCS6*, and *CDKN1A*) showed any significant alterations in the PCa samples (data not shown). Furthermore, the gene expression levels of *HIF1α*, *RELA,* and *BIRC5* showed a positive correlation with the fumarate content (Figure 5B). For confirmation of the clinical relevance of the genes analyzed, we evaluated the TCGA cancer database, which contains 492 PCa and 152 BT samples. This showed a positive correlation between the transcript levels of *Ki67-HIF1α, Ki67-RELA, Ki67-BIRC5,* and a negative correlation between the transcript levels of *Ki67-SERPINB5* (Table S6). The transcript levels of *BIRC5* and *SERPINB5* showed a significant inverse correlation (Table S6). These data indicated that in PCa, a higher proliferation rate is positively associated with an induced HIF1α and NFκB pathway. Furthermore, by analyzing the disease-free survival data in the TCGA cancer database, we ascertained that high expression of *Ki67* was associated with significantly reduced disease-free survival, and that a low *SERPINB5*, together with a high *BIRC5* transcript expression—indicative of an activated NFκB pathway—was associated with even lower survival rates (Figure 5C). As anticipated, patients with higher ISUP 2014/WHO 2016—grade groups showed increased *Ki67* (*p* < 0.0001) and *BIRC5* (*p* < 0.0001) expression, albeit the expression of *SERPINB5* was not significantly different (*p* > 0.05) in the various grade groups. These results suggest that, in prostate cancer, the increase in the fumarate level might trigger carcinogenesis, partly via the HIF1α and NFκB pathways, which have crucial clinical relevance in patients with prostate cancer.

## 3. Discussion

Oncogenic and metabolic transformations are two major prerequisites for tumor development [2]. Mitochondria play a crucial role in the metabolic transformation of PCa [7] and alterations in the TCA and urea cycles located in the mitochondria are pivotal events in PCa tumor initiation [25]. 

Increased levels of fumarate were found in the PCa tissue. Since fumarate plays a pivotal role in many biochemical processes, including TCA and urea cycles, the exact mechanism resulting in such an increase remains to be determined. However, in view of the increase in argininosuccinate, fumarate, arginine, aspartate, and proline, our data suggest that the higher fumarate content in PCa might be replenished through the urea cycle. The theory that fumarate is defined as an oncometabolite is derived from studies that analyzed the consequences of fumarate hydratase deficiency, leading to accumulation of fumarate and tumor progression in various cancers [19]. Fumarate was recently identified as a key player in endometrial cancer progression, enhancing cell proliferation and migration [26]. The oncogenic pathways and their major regulators, which are activated by fumarate, include HIF1α, NFκB, antioxidant response, epigenetic alterations, and protein succination [27]. To ascertain which of the oncogenic pathways could be triggered by fumarate in PCa, we analyzed the gene expression of *HIF1α*, the NFκB subunit *RELA*, and its target genes (*SERPINB5* and *BIRC5*) in extended patient cohorts. In PCa, we observed a higher expression of *HIF1α* and *RELA* and the expression of these genes showed a positive correlation with the fumarate content. NFκB is known to increase the transcript level of the survival protein *BIRC5/survivin* and to reduce the mRNA level of the metastatic suppressor *SERPINB5/maspin* in PCa samples and cell lines, respectively [28,29]. We found an increased expression of *BIRC5* and a decreased expression of *SERPINB5* in the PCa samples and the transcript level of *BIRC5* showed a positive correlation with fumarate content. These results therefore suggest that the increased fumarate content might be responsible for the activation of the HIF1α and NFκB pathway. Furthermore, we utilized the data of the TCGA database; however, these were not adjusted to the clinical parameters. Since the appropriate benign tissue controls are not usually available in the PCa samples, these data tend to represent inhomogeneous cohorts. The data of the TCGA database demonstrated that those PCa patients who showed low *SERPINB5* and high *BIRC5* transcript expression were characterized by a reduced disease-free survival rate, thereby highlighting the clinical relevance of the activation of the NFκB pathway in human prostate cancer. In PCa, the increased fumarate level might induce these oncogenic pathways, which, in turn, could drive cancer progression. The clinical relevance of fumarate was also demonstrated by the studies that analyzed vast amounts of patient material, including samples at different tumor stages. Having analyzed more than 300 PCa samples, McDunn and colleagues observed that a high fumarate content showed a positive correlation with increasing Gleason scores (Gleason 6->Gleason 7a->Gleason 7b->Gleason 8) [30]. Shao and colleagues investigated two independent cohorts consisting of prostate samples with various Gleason scores, and the authors observed a clear positive correlation between the fumarate content and the Gleason scores in both cohorts [15]. During cancer development, the upstream regulator, which could elevate the intracellular fumarate content, was yet to be determined. However, the adaptor protein beta-arrestin 1 (ARRB1) was a possible candidate, since ARRB1 was capable of inducing fumarate level in prostate cancer cells [31].

As already reported [32,33,34], PCa samples showed significantly lower citrate concentrations than the ABT controls. Since the reduced concentration of zinc in PCa could reactivate aconitase activity, the decreased citrate level might be attributed to the metabolic transformation of normal citrate-accumulating cells to malignant citrate-oxidizing cells [35]. A comparative study of high-risk (Gleason score ≥ 8) and low-risk samples (Gleason score = 6) reported an inverse correlation between the level of citrate and Gleason score, indicating that a lower citrate level is associated with PCa development [36]. Moreover, a lower citrate concentration is postulated to be an indicator of cancer aggressiveness [30,37]. The citrate/choline and citrate/lactate ratios also provide valuable information about the energy and tumor status of PCa [38]. As already reported elsewhere [39], in PCa samples, we found decreased ratios of citrate/choline (9.5 ± 4.6 in PCa and 23.2 ± 10.3 in ABT samples, *p* = 0.0039) and citrate/lactate (0.5 ± 0.2 in PCa and 1.1 ± 0.6 in ABT samples, *p* = 0.0195), indicating the altered malignant metabolism of PCa. 

Although the altered citrate concentration was supported by many studies, in human PCa, the reduced concentrations of cis-aconitate and isocitrate are not yet reported in human PCa. Some patients with PCa were characterized by mutations in the isocitrate dehydrogenase (*IDH1*) gene, which encodes the cytosolic enzyme that metabolizes isocitrate [40]. The expression of IDH1 was associated with PCa progression and could be regulated by androgen signaling [41]. Furthermore, alterations in the gene expression level of *IDH2*, which encodes the mitochondrial isocitrate dehydrogenase, were linked to tumor growth or tumor suppression [42]. Further studies are necessary to determine the clinical relevance of the altered cis-aconitate and isocitrate concentrations.

Metabolites involved in the hexosamine pathway were increased in the PCa tissues compared to ABT, which was in line with an earlier study by a Chinese cohort [15]. O-linked beta-N-acetylglucosamine modifications were shown to regulate cancer cell proliferation, survival, invasion, and metastasis [43]. Our data showed that metabolites belonging to glycerophospholipid metabolism were elevated in PCa, as already partly described [16]. Metastatic PCa was characterized by a higher glycerophospholipid signal, when compared to non-metastatic PCa [44]. Many amino acid metabolites showed higher concentrations in PCa tissues (e.g., cysteine, lysine, and branched-chain amino acids), as already partly reported [15,16,45]. The relevance of the altered amino acid metabolism was also underlined by the possibility of using these metabolites as biomarkers for PCa. Higher amino acid metabolite levels were detected in the plasma and serum from patients with PCa than from individuals with benign prostatic hyperplasia [46]. In summary, the higher hexosamine, glycerophospholipid, and amino acid metabolites represent a metabolic pattern, which is specific to malignantly transformed prostate tissue.

One possible limitation of our study is the small sample size of the analyzed cohorts. However, studies involving a higher number of prostate samples that compared PCa to benign controls, found a somewhat similar metabolite pattern [15,16]. Although some studies using LC–MS and GC–MS reported higher concentrations of few urea cycle metabolites in PCa tissue [15,30], our study was, to our knowledge, the first one to report increased aspartate, argininosuccinate, fumarate, arginine, and proline levels, in combination in PCa. Furthermore, the applied CE–MS method enabled us to detect many hydrophilic polar metabolites, which were less well covered by other approaches.

Several drugs are currently shortlisted for PCa treatment. Calcitriol, which is the bioactive form of vitamin D, is characterized by its anti-tumor properties, including induction of cell cycle arrest, apoptosis, inhibition of invasion, metastasis, and angiogenesis [47]. Calcitriol treatment led to decreased fumarate and elevated citrate, as well as isocitrate contents in the PCa cell line LNCaP, which was associated with a lower ATP level [48]. The high fumarate and low citrate and isocitrate levels in PCa observed in our study might therefore indicate a malignant metabolic phenotype. Finally, a reversal of these metabolic disturbances provides us with unique possibilities for future therapeutic approaches.

## 4. Materials and Methods 

### 4.1. Chemicals

HPLC-grade methanol, methyl-*tert*-butylether (MTBE) and acetonitrile used for sample preparation were purchased from Merck (Darmstadt, Germany). The ultrapure water used for preparing the solutions and the mobile phase was purified by a Milli-Q system (Millipore, Burlington, MA, USA). Formic acid (Fluka, Darmstadt, Germany), ammonium bicarbonate (Sigma-Aldrich, St. Louis, MI, USA) and ammonium acetate (Tedia, Fairfield, OH, USA) were used as mobile phase additives. The internal standards were purchased from Isotec (Miamisburg, OH, USA) (leucine-d3, phenylalanine-d5), Sigma-Aldrich (USA) (D-camphor-10-sulfonic acid sodium salt, L-methionine sulfone, succinic acid-13C4, chenodeoxycholic acid-d4, cholic acid-d4, Alanine-d3), Cambridge isotope (Tewkesbury, MA, USA) (tryptophan-d5, FFA C16:0-d4), Avanti lipids (Alabaster, Al, USA) (LPC19:0), and ten Brink (Roden, The Netherlands) (FFA C22:0-d4). 

### 4.2. Human Samples

For this study, we recruited newly-diagnosed PCa patients prior to radical prostatectomy, who had not received treatment before surgery. Tissue sampling was performed by an experienced uropathologist. Both PCa and the adjacent control tissue were immediately snap-frozen in liquid nitrogen and stored at –80 °C. Hematoxylin- and eosin staining was performed on paraffinized samples, for histological confirmation. Histopathological features were assessed and pT- and postoperative ISUP 2014/WHO 2016 grades were determined [23]. Since we were interested in both (i) the metabolite measurements of prostate tissues, which consumed the greater part of the prostate sample material, and (ii) the gene expression level of candidate genes, we analyzed (i) the “mass spectrometry cohort” (Table 1), which consisted of thirteen patients with PCa and paired autologous tumor-adjacent benign prostatic tissue (ABT) samples and (ii) the “extended cohort” (Table 2), including some of the samples of the mass spectrometry cohort extended with new samples, and which contained 21 PCa and 23 benign prostatic tissue (BT) controls. One patient of the mass spectrometry cohort was diagnosed with type 2 diabetes. Informed written consent was obtained from all participants, and the Ethics Committee of the University of Tübingen approved the protocol in accordance with the Declaration of Helsinki. Plasma concentrations of C-reactive protein (CRP) and glucose were measured using ADVIA XPT clinical chemical analyzer (Siemens Healthineers, Erlangen, Germany). Insulin was analyzed using ADVIA Centaur XPT (Siemens Healthineers) and glycated hemoglobin A1c (HbA1c) was measured with the TOSOH G8 HPLC system (Sysmex, Norderstedt, Germany). The ISI Matsuda index was calculated, as reported previously [4,24].

### 4.3. Sample Preparation for Mass Spectrometry (MS) Measurements

For the LC–MS analysis, 80% methanol with internal standards (ISs) was used to extract the metabolites. A total of 10 mg of wet tissue were weighed and placed in a 2 mL tube, followed by the addition of a steel bead and 1 mL extractant. Following homogenization in a TissueLyser (Qiagen, Hilden, Germany) (30 Hz, 1 min × 2) and centrifugation (14,000 g, 10 min, 4 °C), the supernatant was dried in a vacuum centrifuge. 

For the CE–MS analysis, we first depleted lipids using an MTBE-based extraction method that was already described for lipidomics analysis [49]. A steel bead and 400 μL 75% methanol (with ISs) were added to 10 mg wet tissue in a 2 mL tube, then homogenized twice at 30 Hz for 1 min. A total of 1 mL MTBE was added and shaken for 1 h at room temperature, followed by the addition of 250 μL water to induce phase separation. After spinning at 10,000 rpm for 10 min, the lower, lipid-depleted layer was centrifugally filtered through a 5 kDa cutoff filter (Millipore), to remove residual protein before being dried and used for CE–MS analysis.

### 4.4. Liquid Chromatography Coupled with Mass Spectrometry (LC–MS)

The dried samples were reconstituted in 80% methanol for the LC–MS analysis. The chromatographic separation methods are as described in [16]. Metabolites were separated and detected in both electrospray ionization (ESI) positive and negative mode by ACQUITY Ultra Performance Liquid Chromatography (UPLC, Waters Corporation, UK) coupled to a linear ion-trap quadrupole (LTQ) Orbitrap MS (ThermoFisher, Waltham, MA, USA) system. For the ESI positive mode, the ACQUITY UPLC BEH C8 1.7 μm (2.1 × 100 mm) column was implemented. The mobile phases were 0.1% formic acid in water (A) and 0.1% formic acid in acetonitrile (B). The elution gradient was initiated at 5% B for 1 min, was then linearly increased to 100% B at 24 min, maintained for 4 min, and returned to 5% B. For the ESI negative mode, metabolites were separated on an ACQUITY UPLC HSS T3 1.8 μm (2.1 × 100 mm) column. The mobile phases were water with 6.5 mM ammonium bicarbonate (C) and 6.5 mM ammonium bicarbonate 95% methanol solution (D). The elution gradient was as follows—2% D for 1 min, increased to 100% D at 18 min, maintained for 4 min, and returned back to 2% D for post equilibrium. The flow rate was 0.35 mL/min, and the column oven was set at 60 °C for both the ESI positive and negative mode. The parameters of the LTQ–Orbitrap MS were set as follows—for the positive mode, the capillary temperature, 320 °C; sheath gas flow, 40 arbitrary units; aux gas flow, 15 arbitrary units; source voltage, 4.5 kV; source current, 100 μA; capillary voltage, 31 V; and tube lens, 80 V. For the negative mode, the capillary temperature, 325 °C; sheath gas flow, 40 arbitrary units; aux gas flow, 15 arbitrary units; source voltage, 4.0 kV; source current, 100 μA; capillary voltage, 40 V; and tube lens, 122.78 V. The resolution was 30,000. From the paired mass spectrometry cohort, 12-12 PCa and ABT samples were measured using LC–MS in negative and positive mode.

### 4.5. Capillary Electrophoresis Coupled to Mass Spectrometry (CE–MS)

Polar metabolomics profiling analysis was performed on a capillary electrophoresis (CE, G7100A, Agilent, Santa Clara, CA, USA) system, equipped with a 1260 ISO pump (G1310B, Agilent) and a Huber mini chiller (Offenburg, Germany). A fused silica capillary (80 cm × 50 μm i.d.) was used for the metabolite separation. The capillary temperature was maintained at 20 °C. The sample tray temperature was maintained at 5 °C, and controlled by the mini chiller. The mass detector was a time of flight mass spectrometer (TOF/MS) (G6224A, Agilent), equipped with an ESI–MS sprayer kit (G1607A, Agilent). The CE–MS coupling was realized by a coaxial sheath liquid interface. The sheath liquid containing methanol/water (1:1, *v*/*v*) and 0.1 μM hexakis (2, 2-difluoroethoxy) phosphazene was delivered at 10 μL/min. CE–TOF/MS metabolomics analysis of the samples was carried out in both the cation-positive mode and the anion-negative mode. From the paired mass spectrometry cohort, 9-9 and 13-13 PCa and ABT samples were measured using CE–MS in the anion and cation mode, respectively.

### 4.6. Real-Time PCR

Total RNA from the human prostate tissues was isolated using Allprep RNA/DNA/protein kit (Qiagen), in accordance with the manufacturer´s instructions, and cDNA was synthesized (Transcriptor First Strand cDNA synthesis kit, Roche, Basel, Switzerland) [50]. Real-time PCRs were performed from the extended cohort (21 PCa and 23 BT samples) with a LightCycler 480 Probes Master (Roche) with universal probe library (primer sequences), using LightCycler 480 (Roche), as published previously [51]. Delta-delta crossing-point (Cp) values were calculated and the values were normalized to the housekeeping gene ubiquitin c (*UBC*). For real-time PCR analysis, the following primers and probes were applied: *Ki67* 5′-CCAAAAGAAAGTCTCTGGTAATGC and 3′-CCTGATGGTTGAGGCTGTTC (probe nr 39), *HIF1α* 5′-GATAGCAAGACTTTCCTCAGTCG and 3′-TGGCTCATATCCCATCAATTC (probe nr 64), *RELA* 5′-ACCGCTGCATCCACAGTT and 3′-GATGCGCTGACTGATAGCC (probe nr 47), *BIRC5* 5′-CCGCATCTCTACATTCAAGAACT and 3′-GCCAAGTCTGGCTCGTTC (probe nr 43), *SERPINB5* 5′-GCAATGTCCTCTTCTCTCCAAT and 3′-CCAAAGGGTACATCTTTGACATT (probe nr 39), *UBC* 5′-GGAAGGCATTCCTCCTGAT and 3′-CCCACCTCTGAGACGGAGTA (probe nr 11).

### 4.7. Data Pretreatment and Statistical Analysis

The acquired data were transformed to the mzData format for the LC–MS analysis and then imported to the XCMS software for feature extraction and peak alignment [49]. All original data were normalized to the total intensity of metabolite ions of each analyte [52]. For the CE–MS analysis, the data were imported into the Qualitative Analysis Software (B.04.00, Agilent), to extract the electrophoresis spectra of the ISs. Migration time (MT) of the detected metabolites was corrected using the MethodMarker software (HMT, Japan), according to the MT of the ISs. The identification of the metabolites was achieved based on the accurate masses and corrected MT of IS in the HMT database. The peak integration was conducted using the Quantitative Analysis Software with ±20 ppm of the m/z window and ±1.0 min of the MT window. The intensity of each metabolite was normalized to the IS intensity and the tissue weight. Orthogonal partial least square discriminate analysis (OPLS–DA) was performed with the SIMCA 13.0 (Umetrics, Umea, Sweden) software. To determine the differential metabolites, paired, non-parametric, Wilcoxon signed-rank two-sided test was performed, based on the Matlab (MathWorks, USA) program. Metabolite clustering was implemented using the Multi Experiment Viewer (MEV) software [53,54] and the metabolic pathway enrichment was performed on the MetaboAnalyst Web site (http://www.metaboanalyst.ca). Mann-Whitney U tests and Spearman correlations were calculated using the GraphPad Prism 8.1 (USA). Statistical significance was assumed to be *p* < 0.05.

## 5. Conclusions

In summary, our data showed that in human prostate cancer samples, the content of fumarate was increased, which was positively associated with carcinogenic genes.

## Figures and Tables

**Table 1:** Patient characteristics—mass spectrometry cohort.

Patient Characteristics	Mean	Stdev	nr
Age (y)	63	7	13
BMI (kg/m^2^)	26.2	3.3	13
Fasting blood glucose (mg/dL)	102	10	13
Fasting insulin (pmol/L)	86.4	40.6	13
OGTT derived insulin sensitivity index	2.1	0.9	13
HbA1c (%)	5.6	0.2	13
CRP (mg/dL)	0.22	0.53	13
pT-2a			2
pT-2c			8
pT-3a			3
pN			0
ISUP 2014/WHO 2016—Grade Group 2			9
ISUP 2014/WHO 2016—Grade Group 3			4

Abbreviations. BMI: body mass index, CRP: C-reactive protein, HbA1c: glycated hemoglobin A1c, oral glucose tolerance test (OGTT) derived insulin sensitivity index was determined according to Matsuda and DeFronzo [24], nr: number of patients, and Stdev: standard deviation.

**Table 2:** Patient characteristics—extended cohort.

Patient Characteristics	BT	PCa	*p*-Value
Age (y)	62 ± 6	65 ± 7	0.1877
BMI (kg/m^2^)	27.5 ± 3.6	25.7 ± 3.2	0.1352
Fasting blood glucose (mg/dL)	104 ± 10	103 ± 10	0.8203
Fasting insulin (pmol/L)	91.9 ± 39.6	82.4 ± 36.1	0.3786
OGTT derived insulin sensitivity index	2.0 ± 1.2	2.4 ± 1.5	0.3756
HbA1c (%)	5.5 ± 0.4	5.7 ± 0.2	0.1415
ISUP 2014/WHO 2016—Grade Group 2	-	13	
ISUP 2014/WHO 2016—Grade Group 3	-	6	
ISUP 2014/WHO 2016—Grade Group 4	-	2	
ISUP 2014/WHO 2016—Grade Group 5	-	2	
nr	21	23	

## Supplementary Materials

The following data are available online at https://www.mdpi.com/2072-6694/12/7/1814/s1. Table S1: CE–MS anion mode metabolites. Table S2: CE–MS cation mode metabolites. Table S3: LC–MS negative mode metabolites. Table S4: LC–MS positive mode metabolites. Table S5: Selected significant metabolites of the mass spectrometry cohort. Table S6: Correlation analysis of the indicated genes from the human cancer genome atlas.
